# Notes of an European Tour

**Published:** 1845-07-01

**Authors:** F. H. Hamilton

**Affiliations:** Professor of Surgery in Geneva Medical College


### BUFFALO MEDICAL JOURNAL

**Vol. 1.** JULY 1, 1845. **No. 2.**

### Notes of an European Tour. --- No. II.

Communicated for the Buffalo Medical Journal, by F. H. HAMILTON, M. D. Professor of Surgery in Geneva Medical College.

Dear M.

To follow the Parisian Hospital Surgeons and Physicians one must learn to rise early, and fast late. Between 6 and 7 A. M. most of them are at their posts, and until ten or eleven are constantly employed in walking the wards, operating or lecturing. You may well imagine, therefore, with how keen a relish we entertain the first meal: indeed I must confess that the fatigue and depression consequent upon such unusual abstinence, did for a while produce a sensible depression in my medical enthusiasm: a few weeks, however, have rendered me quite tolerant of the custom, and the thoughts of "cafe au lait," and the delicious "omelette," now seldom intrude upon the sanctity of hospital hours.

"*Hotel Dieu*" is to me a venerated name, associated as it has ever been with the memories of Desault, Bichat, Moreau and Dupuytren: and the first morning I spent in Paris was within its wards. Its principal buildings are upon that island of the Seine situated near the center of Paris, and called "La Cite," where, at a remote and unknown period, a wandering tribe of barbarians built their huts and pastured their flocks; dwelling quietly and in peace, until invaded and dispossessed by Julius Cæsar, about 50 years before Christ. Here, close by the Hospital, Cæsar built a palace, which became afterwards a frequent temporary residence of the Roman Emperors: it stood where now stands the gloomy "*Palais de Justice.*" In the year 524 the Romans were in their turn driven out, and Clovis, the first King of the French, enclosed the island with walls. In the following century the Hospital of "Hotel Dieu" was erected just within the southern line of rampart: I believe it therefore to be the earliest Hospital in the world still remaining upon its ancient foundation. Since this early day, however, it has undergone numerous alterations, and by successive Kings, great additions and improvements have been made, until it has become the largest and one of the most healthy Hospitals in Paris. At present it consists of three distinct buildings, two of which stand upon "La Cite," and the third upon the southern bank of the Seine, the two opposite buildings being united by a covered bridge, and a tunnel which traverses the quay.

Hotel Dieu formerly contained 6 or 7,000 beds; the great mortality within its wards, however, induced the authorities to reduce the number of beds to 1,200. In 1825 its mortality was but one in nineteen. The number of annual receptions is now about 11,000, and the average mortality has grown again to one in eight and seventy-two hundredths!

The "Salles" are large and sufficiently airy, and like all similar institutions in catholic countries, are attended constantly by the "Sisters of Charity," whose unwearied devotion to the interests of humanity is ever a subject of surprise and admiration. Those who wait at Hotel Dieu, sixty in number, belong to the order of St. Augustine.

Roux is now the chief Surgeon at this Charity and ranks, since the death of Dupuytren, first in Paris, if not in the world. He is 75 years old and has been in active practice 42 years, yet his vigor of mind and body seem quite unimpaired, and his success as an operator undiminished. Among many other operations, I saw him make a delicate dissection of a tumor from the neck of an old man. The operation was necessarily tedious and required great care, yet it was accomplished in a steady and skillful manner. Before commencing the operation, M. Roux adverted to the occasional admission of air into veins, during operations in this region — one instance of which occurred in his own practice in 1833, while he was surgeon at La Charite. He cut (accidentally of course) the internal jugular entirely across, and immediately the patient, (a girl,) sank back and seemed to have expired. M. Roux cast a ligature about the base of the tumor, not yet entirely dissected out, and left it to come away spontaneously; she revived shortly, lived seven days, and then died comatose. Parisian surgeons dare thus speak publicly of their own surgical accidents; a species of courage and honesty, which, with us at home, would ensure a prosecution for malpractice, if not total loss of reputation. Roux has opened at other times, by accident, the subclavian vein, the axillary, and the internal jugular, and Velpeau declares openly that he has "often" cut the internal jugular.

Roux occasionally *experiments* in union by "first intention" after amputations, and I have seen several snccessful experiments of this kind in his wards: but I should do him great injustice, to say that he has yet adopted this *English* principle as an established practice.

The other surgeons to Hotel Dieu, are Breschet, and Blandin. The former is author of several essays upon comparative Anatomy: and he has now in progress a large work on the Anatomy, Physiology, and Pathology of the venous system. He has been 32 years in practice, but has never acquired great celebrity as a surgeon, neither is he with the students a favorite. Blandin is the youngest of the three. His manners are pleasant and unassuming; his habits industrious, and his skill and success as an operator are fully admitted. I remember now to say, that he exsects enlarged tonsils, as do most Parisian surgeons, with a common bistoury and forceps; I still however should give the preference to those well constructed mechanical instruments in use with us; since, while they are equally or more safe, they are more expeditious and less painful. I have seen M. Blandin make of this operation, so simple with us, a tedious and bloody affair.

I cannot avoid giving you here one illustration of union by "second intention" after amputation, as a specimen *en passant* of French Surgical *Therapeutics*. A poor fellow had his leg badly crushed, which M. Blandin amputated the same day above the knee. He made the circular incisions, and when the limb came to the floor, every one exclaimed "admirable." The vessels being secured, a piece of perforated linen, smeared with cerate was placed over the whole surface of the wound,and above this a large quantity of charpie. The edges of the gaping integuments and muscles were then gently approximated, with bandages, &c. Thus dressed, literally *inside* and *out*, he was laid in bed. A few days after, the wound was discharging copiously; irritative fever and night sweats had supervened; and the patient was sinking rapidly. In a few days more he was carried to the "dead room," and M. Blandin himself pronounced the verdict; "unfortunately excessive supuration having occurred, has proved fatal." I added mentally "as a consequence of a charpie surfeit." More of this anon.

The Physicians at H.D. are all, I believe, veterans in practice, and their teachings I regard as almost oracular. M. Husson the Broussaist, and Recamier, have both practiced nearly half a century.

Magendie is the famous Physiologist and "Vivisector," who at one time created no little excitement among the English Philanthropists, because he opened a *rabbit* alive, to benefit his fellow-men, while tender-hearted John Bull was engaged in the innocent pastime of persecuting a *hare* to death for "sport." Notwithstanding the many vivisections practised upon him by his enemies, he still lives and labors at Hotel Dieu.

Rostan, who holds that asthma is dependent upon aneurism of the heart, and the author of an excellent article on ramollissement of the brain, and Chomel, most known through his general pathology and treatise on typhoid fever, are among the physicians. The remaining three are Honore, Caillard and Gueneau.

Dark and frowning over the walls of Hotel Dieu, stand and have stood for centuries, the towers of "Notre Dame:" the great cathedral, about which is woven so much of the history and romance of Paris; which has witnessed the offerings of so many penitent hearts—the burnings of so many heretics and sorcerers in its open "parvis,"—the coronation of so many Kings—which has witnessed, also, the installation of a courtezan as the "Goddess of Reason," upon the very altar of the True God, and the elevation of the "vulgar Corsican" to the throne of an Emperor, in the presence and by the forced consent of the Supreme head of the Catholic Church.

I have neither taste nor ability for architectural descriptions: the anatomy of Burke I propose to give you when I visit Edinburgh, but the anatomy of "Notre Dame" I must leave for others. But I am intelligible to you when I say, that as it is one of the oldest, so it is the purest specimen of ancient pointed gothic in Paris. The style is massive and gloomy, yet simple and majestic, and as I enter its vast and sounding aisles, cold and dimly lighted, a deep and solemn awe pervades me; an impression which is often not a little deepened by the broken and half smothered sobs at the confessionals, and the low murmuring monotone of the Priest. This Church was once rich in relics, but many of these were destroyed during the "Reign of Terror" and in the "emeute" of 1831. In the chapel of "St. Julien the Poor," may, however, still be seen several gilt busts, which enclose some of thc precious remains of the eleven thousand virgins who sufferred martyrdom with St. Ursula, at Cologne, in Germany. The number seems large, yet with regard to relics, I have made a vow, never to entertain scepticism.

From one of the towers of Notre Dame, an excellent view of the city is obtained, and in company with four others, I climbed its four hundred steps. Five is an unusual number to be admitted to the towers at one time, and the old lady who stood at the tower entrance below, hesitated to allow so large a party to pass. The reason assigned is not quite intelligible—that like the "fire tower" in London, this spot has been selected by certain amateurs in suicide, from whence to make the death-leap. We suppose that our good-natured and non-suicidal countenances, with a *small gratuity*, secured for us admission.

Near the top we stopped for a moment, to examine "Emmanuel-Louise-Therese," so baptised by Louis the 14th and his queen Therese; weighing 32,000 lbs., and its tongue 976 lbs. It is moved by four ropes, and requires sixteen men to make it speak. In its hoarse tocsin notes, therefore, the people are constantly reminded of the pious devotion of "La Grand Monarque," the slave of his minister and two mistresses. He died of "gangrena senilis," induced doubtless by his intemperance and debaucheries during life.

Going to and returning from Hotel Dieu, we always stop at "La Morgue." It is the "morning gazette" for all who pass, or reside in this neighborhood, the "tragic theatre" for La Cite. You have two opposite points from which you may obtain a complete moral panorama of Paris, the Foundling Hospital, and La Morgue. La Morgue, (the bone house,) is a small stone building, not many steps from Hotel Dieu, and close upon the banks of the Seine. Here are brought, for recognition, the bodies of all the unknown, who are found dead. On the right as you enter, is the office of the Registrar; a little beyond is a small room with a copper covered dissecting table, where inquests are made The hearse room and cleansing room are next. To the left, separated from the spectators hall by glass windows, is the "chamber of the dead," where, stretched upon inclined planes, stripped of all clothing except a loose partial covering of oil cloth, they are permitted three days to "lay in state." Water is constantly dripping from small tubes upon their bloated and discolored faces, sometimes horribly mutilated and disfigured. Above and around hang the clothes—a laborers blouze and check shirt; a bourgeois's broadcloth coat; a neat gown made in the latest fashion—for a Parisian woman would die decently; a student's velvet embroidered cap; an infant's swaddling clothes. In these mournful testimonials, the spectators read the age, sex, and rank, possibly even the name, of each.

Above, I am told, the family of the "Morgeur,"the Charon of the place, resides ; and he has a daughter who entertains herself, and the dead who live below, with cheerful music.

300 is the annual average of bodies, mostly *suicides*, brought to the Morgue, five-sixths of whom are males, and yet these by no means comprise all the suicides of Paris. I have no means of ascertaining the exact annual number, but they are admitted to be very numerous. Certainly vastly more numerous in proportion, than in any city or portion of the United States. I protest, therefore, against the assertion of Falret, principal physician at Salpetriere, that "republican forms of government favor the development of mental alienation, and consequently suicide."—

It was not during Republican Rome, but under the despotism of its Emperors, that suicides became common. Switzerland is remarkable for the infrequency of its suicides—while England, France and Germany present large and increasing catalogues. The astonishing increase of suicides during the French revolution, discloses, not the effect of republican institutions, but rather the effect of *no* institutions—of anarchy, tyranny, and bald infidelity. To these causes should be traced the 1300 suicides committed in the single town of Versailles, during the year of the "Reign of Terror"—a fact unparalleled in the history of the civilized world.

"La Charite," No. 45, rue Jacob, was founded in 1613, by Maria de' Medicis, who, so unequal are the rewards of this life, died poor and deserted, in an obscure house of Cologne. Her heart is now honored with a burial in the great Cathedral of Cologne near the shrine of the "Three Wise Men" who visited first the Holy Manger.

La Charite contains 530 beds, and receives annually an average of 8000 patients; mortality, 1 in 10,40 —Velpeau, the great anatomist and miracle of industry, is the principal surgeon. You have heard that Parisian Surgeons deride Velpeau's claim to eminence as an operator. They acknowledge his industry and zeal, and intimate, that with such qualities he might have excelled as a "blacksmith," or "farrier," had he never left the calling. I believe these intimations ungenerous and unjust; he certainly is not a rapid, yet for the same reason is he, a more safe operator. I give you his maxim in his own words. "Je sais maintenant que sur l'homme vivant, il import moins d'agir vite que d'agir convenablement." And it might be well for some unfortunates had French Surgeons, generally more of Velpeau's caution.

I have seen Velpeau operate twice for cancer, which, with Roux, Blandin, Gerdy,Lisfranc, and others, he believes originally a *local* affection and only consecutively constitutional. He therefore removes many cases of scirrous, and open cancer, which American Surgeons would hesitate to touch. But my attention has been especially attracted to his treatment of *all* simple fractures by "dextrine;" constituting a species of the immoveable apparatus. Whether "dextrine" possesses any decided advantages over the "starch" of M. Suetin, I am not prepared to say—it is certainly liable to most, if not all the objections which have so frequently been made against the latter, and indeed against every other mode of close immoveable apparatus. With regard to either method as an indiscriminate practise, I have not changed my often expressed opinion, that none of them will abide the test of time. Even Velpeau, if he lives many years, will be forced to recall his abused and rejected splints, extension and counter-extension, &c. I have not, it is true, seen in his wards an unfortunate result; but I am assured by students of intelligence and veracity, that in their observations, such cases have occurred under his own eye, and which timely knowledge of the actual condition, and application of proper apparatus might have prevented—these cases are not given to the world. He still adheres to his method, of treating Hydrocele, by injection of tincture of iodine. He admits that he has by accident, occasionally thrown it into the cellular texture, but no sloughing has ever followed. He varies the strength, diluting it always with water, injects it cold and immediately withdraws it. I saw Liston in London using and reccommending the same, and I think it may now be held an established practice in England, France and America. Nitrate of silver, as an application to inflamed eyes, in their various stages, either as an unguent, wash, or in substance, constitutes with him almost a catholicon, and I am pleased to find that I have been riding my "hobbie" with so distinguished a man.

M. Gerdy is the second Surgeon to LaCharite, and of nearly the same age with Velpeau, yet much less distinguished. As a writer however, upon various Anatomical, Surgical and Medical topics, he is well known. He has just now in press and partly issued a large work entitled "Physiologie Medicale."

Here also in the Medical wards are the two distinguished pathologists, Andral and Cruvelhier. Cruvelhier, has a mild, intelligent and attractive, yet not handsome face, and is with me a favourite. Rayer is fat as an Alderman, and carries himself about the wards with such a distinguished air of ease and comfort that you would scarcely take him for the student which his late writings have shown him to be. In diseases of the kidneys and skin he is acknowledged authority. Bouillaud, a Broussaist, Phrenologist, and author of a work on diseases of the heart, and Fouquier have, also, each a service at La Charite.

Returning from La Charite, I have often been attracted to the ancient church of "St. Germain des Pres," or the "Golden Basilic;" the Abbey church of the most ancient monastic establishment of Paris, having been founded in the year 560. It contains the tombs of Descartes, Mabillon Mountfaucon, also of Casimir, king of Poland, who in 1668, abdicated his throne to become abbot of this monastery. Adjoining is still seen the prison of the abbey, blackened, and time-scathed; now converted into a military guard house.

